# Impact of Intraoperative Factors on the Development of Postpartum Septic Complications

**DOI:** 10.3390/medicina59091637

**Published:** 2023-09-10

**Authors:** Diana Andzane, Anna Miskova, Antra Krone, Dace Rezeberga

**Affiliations:** 1 Riga Maternity Hospital, Miera Street 45, LV-1013 Riga, Latvia; anna.miskova@rsu.lv (A.M.); antra.krone@rdn.lv (A.K.); dace.rezeberga@rsu.lv (D.R.); 2 Gynaecology Clinic, Riga East Clinical University Hospital, Hipokrata Street 2, LV-1079 Riga, Latvia; 3 Department of Obstetrics and Gynaecology, Rīga Stradiņš University, Miera Street 45, LV-1013 Riga, Latvia; 4 Department of Clinical Skills and Medical Technologies, Rīga Stradiņš University, Anninmuizas Bulvaris 26a, LV-1067 Riga, Latvia

**Keywords:** antibacterial sutures, triclosan, caesarean section, postpartum endometritis

## Abstract

*Background and Objectives:* Triclosan-coated sutures (antibacterial sutures) can reduce the risk of postoperative surgical site infection. This study aimed to investigate the effect of intraoperative factors, including antibacterial sutures, on the risk of postpartum septic complications. *Materials and Methods:* The prospective study included patients who underwent caesarean section. The exclusion criterion was chorioamnionitis. The investigation group patient’s (*n* = 67) uterus and fascial sheath of the abdominal wall were sutured with triclosan-coated polyglactin 910 sutures during surgery. The control group consisted of 98 patients using uncoated polyglactin 910 sutures only. The patients were contacted by phone after the 30th postoperative day. *Results:* No significant difference was found between the investigation group and the control group in the development of postpartum endometritis (11.7% in the investigation group vs. 8.4% in the control group, *p* = 0.401), wound infection (6.3% vs. 3.6%, *p* = 0.444) or patients experienced any septic complication (15.9% vs. 12%, *p* = 0.506). Postpartum endometritis was more common in patients who underwent instrumental uterine examination during the surgery (23.8% vs. 18%, *p* = 0.043). A moderately strong correlation was found for haemoglobin level on the third–fourth postoperative day with the development of postpartum septic complications, *p* < 0.001, Pearson coefficient −0.319. Post-caesarean delivery septic complications were not statistically more common in patients with blood loss greater than 1 L. The incidence of post-caesarean endometritis was 13.4%, and wound infection was 4.8% in this study’s hospital, having five to six thousand deliveries per year. *Conclusions:* Using antibacterial sutures during caesarean section does not affect the incidence of postpartum septic complications. Instrumental uterine examination during caesarean section increases the risk of post-caesarean endometritis and is, therefore, not recommended. Haemoglobin level on the 3rd–4th postoperative day, rather than the estimated blood loss during surgery, affects the development of postpartum septic complications.

## 1. Introduction

A caesarean section is the most common surgical procedure performed on women [1], and it is also a significant postpartum risk factor for infection [2]. The caesarean section increases the incidence of endometritis and wound infection to 16–17% [3] and increases infection-related mortality by 25 times [4,5]. El-Achi et al. found in their study that repeated hospitalisation due to infection is more common after an emergency caesarean section (88%) than after a scheduled surgery (12%) [6]. The authors explained that before emergency surgery, amniotic fluid leakage occurs more often, after which the amniotic sac’s sterility is lost, contributing to the contamination of the uterine cavity and the wound. Their second explanation is the imprecise observation of the exposure time for preoperative skin disinfection with an antiseptic solution before emergency surgeries. A significant cause for the development of infection in patients after caesarean section is a large part of devitalised tissue, as well as foreign bodies (suture material), representing a favourable environment for contamination and development of infection [2].

**Figure 1:**
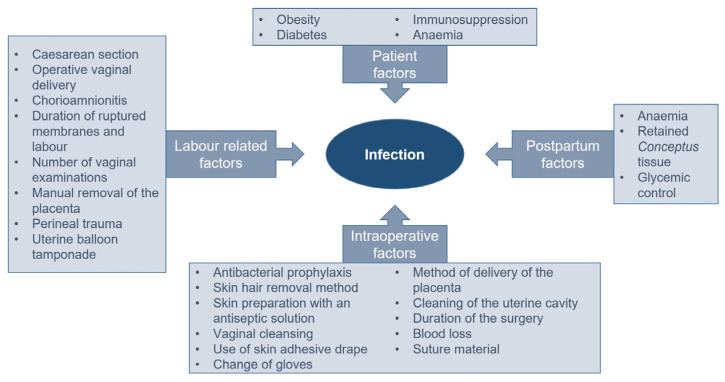
Risk factors for postpartum septic complications.

Over recent decades, multiple factors, which affect the risk of developing septic complications after vaginal delivery and caesarean section operation and which can potentially be affected, have been identified and shown in Figure 1. One group of such factors includes intraoperative factors. Multiple approaches for reducing the adverse intraoperative factor effects on the development of infections are being researched, including suture material.

A surgical suture used during surgery is a foreign body that remains in the body and may affect the risk of infection. Bacteria colonising the suture material form a biofilm on its surface that is resistant to antimicrobial agents and the human immune system. The formation of biofilms increases the possibility of wound infection [7]. Suture material impregnated or coated with a broad-spectrum biocide can provide a local concentration of antimicrobial agents around the sutures, inhibiting the formation of biofilms and the development of infection [8]. Triclosan-coated suture material has been approved by the US Food and Drug Administration since 2002 [7]. Triclosan (polychlorophenoxyphenol) is a broad-spectrum antiseptic agent with antibacterial and antifungal activity, which has been used in toothpaste, soaps, skin treatment agents, etc. for many years [7,8,9]. Triclosan inhibits the enoyl-acyl carrier protein reductase, which inhibits bacterial fatty acid synthesis, cell membrane construction, and reproduction [8,10]. It has not been found to have carcinogenic, teratogenic, or mutagenic effects and has a shallow risk of resistance development [7,9,10]. Triclosan-coated suture material effectively inhibits bacterial colonisation of sutures by up to 66% in vitro and in vivo studies [7,11]. Such antibacterial sutures are widely researched in multiple surgery sectors—colorectal, gastrointestinal, cardiovascular, orthopaedics, and pediatric surgery [8], where their efficiency in reducing wound infections has been confirmed [7,10,12]. Extensive meta-analyses have been performed. Ahmed et al.’s meta-analysis conducted in 2019 summarises 25 randomised trials with 11,957 participants who underwent any surgery [7]. The authors conclude that using triclosan-coated sutures reduces the risk of wound infection (relative risk (RR) 0.73, 95% CI 0.65–0.82). Subgroup analyses have revealed that these sutures are effective only in clean and contaminated surgeries but not in clean–contaminated and dirty surgeries. The authors explain their results in the subgroup of dirty surgery with the low number of patients in this subgroup (102 patients in the antibacterial suture group and 105 patients in the standard suture group) but do not comment on the ineffectiveness of antibacterial sutures in clean–contaminated surgery. Therefore, this moderate-quality evidence recommends using antibacterial sutures in clean and contaminated surgeries [7]. Another large, randomised trial, known as the FALCON trial, with 5788 participants, was conducted in low- and middle-income countries, and the results were published in 2021. This study found no superiority of triclosan-coated sutures in reducing surgical wound infection in clean–contaminated, contaminated, or dirty operations [13]. World Health Organization (WHO) guidelines recommend using antibacterial sutures to prevent post-surgical wound infection, irrespective of localisation [14]. However, these surgical sutures practically have not been researched in obstetrics. Possibly, their effect is similar to the results of other surgical operations, and the use thereof can reduce the development of infection after caesarean section surgery. The risk of infection after caesarean section is affected by many other important factors, for instance, risk factors of the birth process. Is the antiseptic effect of antibacterial sutures sufficient to influence the development of infection after a caesarean section as well? This study aimed to investigate the impact of intraoperative factors, including antibacterial sutures, on the risk of postpartum septic complications.

## 2. Materials and Methods

### 2.1. Design of the Research and Population

A prospective study was conducted at Riga Maternity Hospital from 20 July 2018 to 21 December 2021. Patients who agreed to participate in the research and had the second-category (urgent), third-category (unscheduled), or fourth-category (scheduled) caesarean section operations were included in this study. All patients signed an informed consent form before the procedure and were randomly included in the investigation (antibacterial sutures) and control groups. Caesarean-section patients of the first category were not included in this study due to insufficient time to explain the nature of this study and obtain consent. Chorioamnionitis was an exclusion criterion. The investigation group patients’ uteruses and abdominal wall fasciae were sutured with triclosan-coated polyglactin 910 sutures during surgeries and the other layers—with uncoated polyglactin 910 sutures. In the control group patients, polyglactin 910 sutures without coating were used to suture the uterus and layers of the anterior abdominal wall. Following local medical protocols, patient preparation for surgery and postoperative care were the same for both study groups.

By local medical protocol, the surgical site hair was removed using a trimmer. Antibiotic prophylaxis was administered 30 min before skin incision with cefazolin 2 g i/v or ampicillin 1 g i/v if it was performed due to positive B group haemolytic streptococcus in the vaginal smear. In the second and third-category caesarean section patients, presurgical cleansing of the vagina with povidone-iodine was performed. The chlorhexidine alcohol solution was used to prepare the skin of the surgical site. Adhesive drapes were used to cover the sterile area of the operation site. The modified Misgav Ladach technique was used for caesarean section at this study’s hospital. The modified points included blunt separation of the fascia after transverse incision of 2 to 3 cm, two layers of suturing of low transverse uterine incision, and closing the skin by subcuticular suturing. The surgeons were free to perform additional manipulations if they considered them necessary. Before the anterior abdominal wall was closed, the operator and the assistant changed gloves to new, sterile ones. In the post-surgical period, the patients were observed at the hospital inpatient until at least the 4th post-surgical day. The wound dressing was removed from the surgical wound 24–36 h after the operation. On the third and fourth post-surgical day, the concentration of C reactive protein (CRP) in the blood plasma, blood haemoglobin level, and white blood cell (WBC) count were determined in all patients. Abdominal pain was assessed according to the Visual Analogue Scale (VAS). After the discharge, the patients were contacted by phone after the 30th post-surgical day. If the patient had any health problems within 30 days after giving birth, detailed information on the healthcare facilities visited, diagnoses, and treatment received were collected.

About 20% of patients developed infectious complications after caesarean section at this study’s hospital or after discharge before the start of this study. To detect a reduction in post-caesarean septic complications with a two-sided 5% significance level and a power of 85%, a sample size of 86–99 patients per group was necessary, given an anticipated dropout rate of 15%. For sample size calculation, the www.sample-size.net (accessed 4 January 2023) webpage was used.

To maintain a 1:1 ratio of participants in the groups, for allocation, alternation was used—the first patient was included in the investigation group, the second in the control group, the third in the investigation group, etc. Informed consent and allocation of the patients were ensured by the principal investigator, who informed the operating nurse who selected the respective suture material. Following her duties, the operating nurse filled out the equipment protocol used during the operation, including the type of suture material. Consequently, the surgeon and postpartum care staff could see the suture material used in the protocol. Post-discharge care providers and enrolled patients were blinded. Outcome adjudicators were unblinded.

Before the beginning of this study, permission was obtained from the Rīga Stradiņš University Research Ethics Committee (Nr. 5/31 May 2018).

### 2.2. Interpretation of Results

Endometritis was diagnosed clinically by detecting at least two of the three following signs: (1) body temperature above 38 °C; (2) pain or tenderness of the abdomen or uterus in the absence of other causes thereof; (3) purulent discharge from the uterus.

The European Centre for Disease Prevention and Control recommendations defined wound infection. 

Septic complications were defined as any infection-related complications in women, including endometritis and wound infection. If a patient developed endometritis and wound infection, it was considered one case of septic complications.

### 2.3. Methods of Statistical Analysis

The obtained data were processed with MS Excel 2111 and IBM SPSS Statistics 22 software. Kolmogorov–Smirnov test was used to assess the distribution of quantitative data (age, time of operation, blood loss, CRP, WBC count, etc.); if these data did not conform with the normal distribution, the Mann–Whitney U test was used for further analysis. Meanwhile, if the normal distribution was detected, the two-tailed Student’s *t*-test for independent samples was used to analyse two groups; for more than two groups, analysis of variance (ANOVA) was used. A chi-square or Fisher exact test was used to statistically assess qualitative data (endometritis, wound infection, blood loss of more than one litre, etc.). The correlation was considered weak if the Pearson correlation coefficient was <0.3; medium-strong if the correlation coefficient was 0.3–0.5, strong if the correlation coefficient was −0.6–0.8, and very strong if the correlation coefficient was >0.8.

The result was considered statistically significant if the confidence level was *p*-value (*p*) < 0.05.

## 3. Results

**Figure 2:**
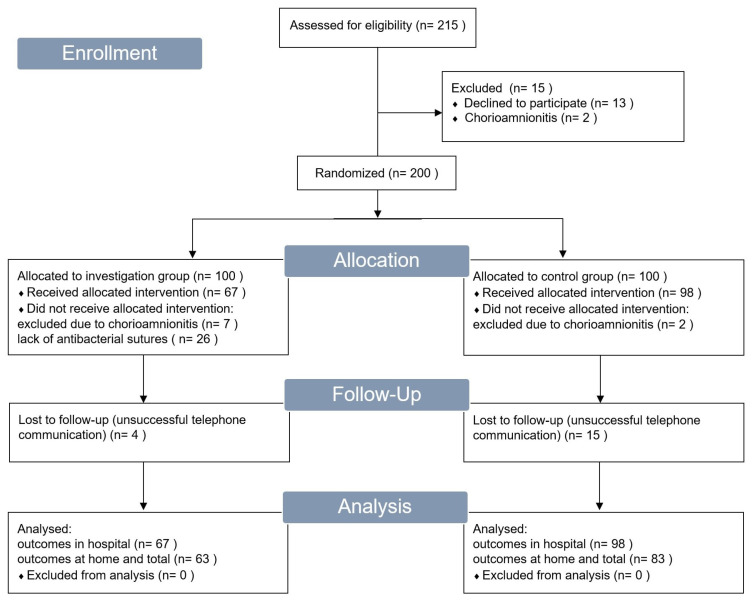
Flowchart of this study’s population.

Two hundred patients were enrolled in this study. Chorioamnionitis was an exclusion criterion, and 11 patients conformed to this criterion. Nine patients developed chorioamnionitis after inclusion into the research and allocation and were prescribed antibacterial therapy for at least three days after childbirth. Therefore, they were excluded from this study. Near the end of this study, the antibacterial sutures suitable for use in this study ran out, and the supply of new sutures was not possible. Therefore, the last 26 patients allocated to the investigation group did not receive the intervention or participate further in this study. As a result, there was an unequal number of participants in the investigation and control groups. There were 67 patients in the investigation group and 98 patients in the control group instead of 100 participants in each group. Thus, 165 patients continued participation in this study and were subject to observation and analysis. The communication and data collection process was successful for 146 (88.5%) women. This study’s flowchart is visualised in Figure 2.

The investigation and control group patients were compared by general status, medical history, pregnancy, and labour parameters. Any of the parameters found no statistically significant difference between the groups. The comparison has been reflected in Table 1. One patient without antenatal care participated in the investigation group, but there was no statistically significant difference between the groups, *p* = 0.414. The investigation and control groups contained patients in whom group B haemolytic streptococcus culturing was not performed. There were nine such patients in the investigation group, while in the control group—13, which is not a statistically significant difference, *p* = 0.235. For patients who had parturition, the duration of labour in the investigation group on average lasted for 9 h 48 min ± 54 min, while in the control group—9 h 24 min ± 54 min, *p* = 0.666. Slightly more than one-half of the patients in this study had ruptured amniotic fluid membranes before caesarean section, where the membranes ruptured spontaneously, or an amniotomy was performed. Out of these, the pre-labour rupture of membranes occurred in 12 (34.3%) investigation group patients and 24 (40.7%) women in the control group, *p* = 0.538. In the investigation group, the rupture of membranes generally lasted for 9 h 54 min ± 5 h 46 min, while in the control group, it was 8 h 42 min ± 7 h 38 min, *p* = 0.407.

**Figure 3:**
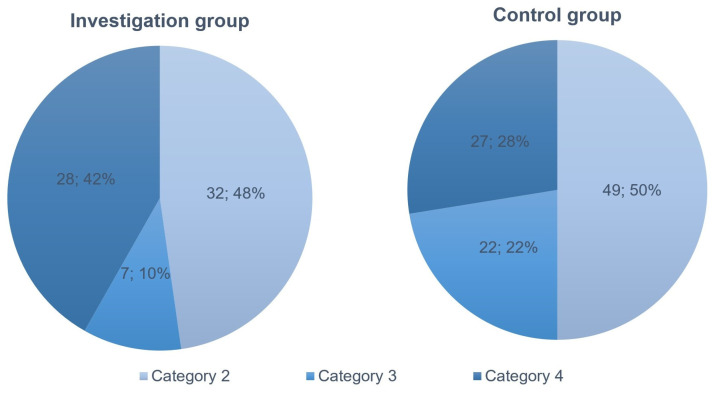
The investigation and control group patients were distributed by caesarean section category, *p* = 0.057.

The distribution of investigation and control group patients by category of caesarean section did not differ statistically significantly, as demonstrated in Figure 3. In both groups, a second category caesarean section was performed in almost one-half of the patients.

Intraoperative factors were considered, which could affect the risk of developing postpartum septic complications. The groups were statistically significantly similar in terms of operation time, blood loss, and frequency of instrumental revision of the uterus, while in the investigation group, subcutaneous suturing was performed more frequently (see Table 2). The frequency of suturing the uterine visceral peritoneum, parietal peritoneum, and rectus abdominis muscle was equal in both groups. Before the operation, one investigation group patient and two control group patients did not receive perioperative antibacterial prophylaxis, *p* = 1. Additional manipulations, which were performed in nine patients of the investigation group and eight patients of the control group during the caesarean section, included bilateral salpingectomy, tubal ligation, ovarian cystectomy, excision of para-ovarian cyst, myomectomy, and ovariectomy.

The post-surgical period did not differ between the groups (see Table 3). Ultrasound examination (US) in the postpartum period was performed on 30 patients of the investigation group and 42 patients of the control group. Among these, the normal US finding was registered in 19 (65.6%) investigation group participants and 27 (81.8%) control group participants. These differences were not statistically significant, *p* = 0.143. The most common US findings included subcutaneous haematoma, ligatures with a coating of fibrine, and uterine subinvolution.

Septic outcomes of post-caesarean section surgery were known to all patients by their discharge date. After discharge, septic outcomes were determined and only calculated for the patients whose successful postdischarge communication was established—63 patients of the investigation group and 83 patients of the control group. Septic complications that developed in the patients included in this study were endometritis, wound infection, and sepsis. None of the patients was diagnosed with an infection of other localisation, for instance, pneumonia, urinary tract infection, mastitis, etc. The frequency of development of septic complications in the investigation and control group was similar both in the hospital and at home after discharge. All septic outcomes in the post-surgical period are reflected in Table 4. One patient in the investigation group developed E. coli sepsis. The recovery of the patient was achieved without surgical manipulations. Two investigation group patients developed recurrent endometritis at home and were treated in an outpatient setting. In both groups, some patients sought help at a medical institution. Still, non-infectious complications were detected in these patients: seroma—in three patients; uterine subinvolution—in one patient; and metrorrhagia—in two patients. In total, 14 out of 15 endometritis cases that developed in all patients included in this study were diagnosed before discharge, and only three were in outpatient care; two were relapses. Meanwhile, wound infection most frequently developed at home after discharge—two cases before and five cases after discharge. Among all wound infections, five were superficial and two—deep.

**Figure 4:**
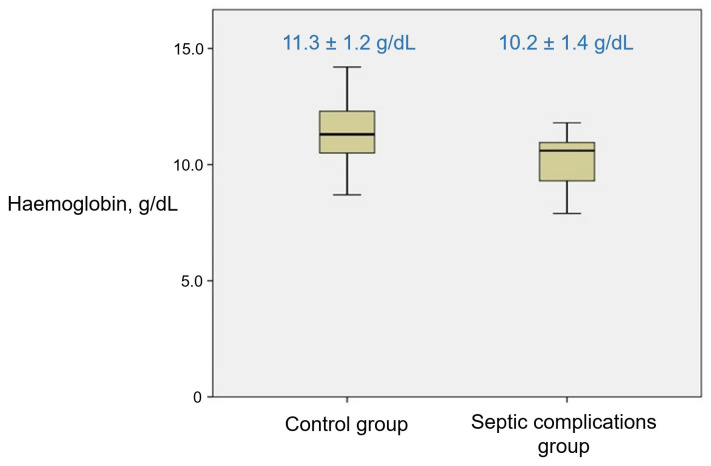
Haemoglobin level on post-surgical day 3–4 in patients with and without septic complications; *p* < 0.001; Pearson coefficient −0.319.

The effect of other intraoperative factors on the development of post-surgical endometritis was calculated in patients with known post-discharge outcomes (146 patients). The results are reflected in Table 5. The duration of the operation did not demonstrate a statistically significant association with the risk of developing septic complications, *p* = 0.183 (Pearson coefficient 0.111). Blood loss that was estimated during the operation was also not statistically significantly associated with septic complications; however, a medium–strong correlation of haemoglobin level on a post-surgical day 3–4 with the risk of developing septic postpartum complications was detected, *p* < 0.001 (Pearson coefficient −0.319) (see Figure 4). The haemoglobin of patients with septic complications averaged 10.2 ± 1.4 g/dL, while patients without septic complications had a haemoglobin level of 11.3 ± 1.2 g/dL. Endometritis was most diagnosed in patients with instrumental revision of the uterus during the surgery. This procedure was performed in 13.9% of all operations of this study. The frequency of endometritis in patients with and without instrumental revision of the uterine cavity during caesarean section can be seen in Table 5. No statistically significant difference in the frequency of developing wound infection between patients with or without intraoperative risk factors was detected. Calculating the effect of intraoperative factors, such as the method of hair removal, type of skin disinfection agent, cleansing of the vagina before the operation, and changing gloves during the surgery, was unsuccessful because joint tactics were used for all study patients. The placenta delivery method was not recorded for this study’s patients. Suturing the uterine visceral peritoneum, parietal peritoneum, and rectus abdominis muscle did not lead to a statistical increase in the development of septic complications.

The frequency of septic postpartum complications was also calculated for parturition factors—preterm birth, body mass index (BMI) ≥ 30 kg/m^2^, patient in labour, the second stage of labour, duration of birth ≥ 12 h, presence of ruptured membranes before caesarean section, pre-labour membrane rupture, ≥5 vaginal inspections and gestational diabetes. A statistically significant increase in the development of postpartum complications was observed in patients who had been in labour before surgery: 76% (20) versus 7.9% (6), *p* = 0.034 and in patients with gestational diabetes 28.6% (6) versus 11.2 % (14), *p* = 0.043. Endometritis more commonly occurred in patients with a duration of ruptured membranes: ≥12 h, 14.8% (12) versus 4.6% (3), *p* = 0.044. Wound infection most occurred in patients who had been in the second stage of labour, 23.1% (3) versus 3% (4), *p* = 0.016, and in patients in whom five vaginal examinations were performed, 10.2% (5) versus 2.1% (2), *p* = 0.043.

**Figure 5:**
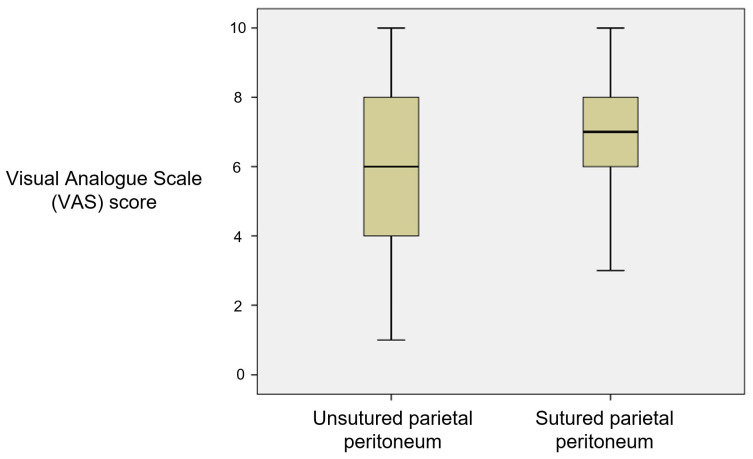
The relationship between suturing of the parietal peritoneum and pain on the 1st postoperative day, *p* = 0.047, Pearson’s coefficient 0.155.

BMI demonstrated a weak but positive correlation with more severe pain on the fourth post-surgical day, *p* = 0.006 (Pearson coefficient 0.215), and a weak but positive correlation with CRP, *p* = 0.01 (Pearson coefficient 0.199). Suturing of the parietal peritoneum weakly but statistically significantly correlated with pain during the first post-surgical day, *p* = 0.047 (Pearson coefficient 0.155) (see Figure 5). No correlation between the suturing of other layers of the anterior abdominal wall and pain intensity during the post-surgical period was detected.

The average levels of CRP in the investigation and control group on the third–fourth post-surgical day did not differ. It was observed that CRP poorly and negatively correlates with haemoglobin levels, *p* = 0.003 (Pearson coefficient −0.233).

**Figure 6:**
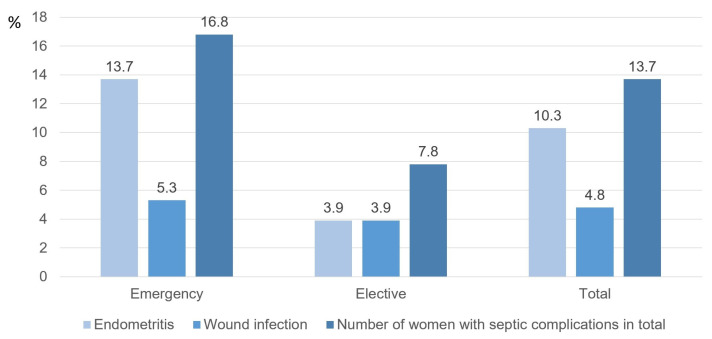
The frequency of development of postpartum septic complications according to the degree of urgency of caesarean section.

The total endometritis rate at this study’s clinic was 10.3 %, and 8.5 % was detected before discharge. The frequency of developing septic postpartum complications according to the urgency of caesarean surgery is reflected in Figure 6.

## 4. Discussion

The development of septic complications in patients where triclosan-coated surgical sutures were used was negligible compared with standard sutures without coating. Therefore, it did not affect the development of septic complications in patients undergoing caesarean section. This finding disproves the set hypothesis. These results differ from research conducted in other areas of surgery, where the positive effect of antibacterial sutures was proven in a high-income country [7,10,12]. However, in the subgroup of clean–contaminated surgeries of Ahmed et al., a large meta-analysis which categorised caesarean section surgery, the use of antibacterial sutures proved to be inefficient [7]. The randomised FALCON study in the countries with a low to medium income level showed that the use of antibacterial sutures for the closure of abdominal wall fascia did not reduce the risk of infection of the surgical wound neither in the clean–contaminated nor the contaminated or dirty surgeries [13]. The authors admitted the unreasonably higher costs of this method and did not recommend the routine use of antibacterial sutures.

The FALCON study only included abdominal surgeries, where, in the subgroup of clean–contaminated operations, 54% (1618 patients) had caesarean sections, and 66.9% were urgent [13]. Most caesarean surgeries in our study belonged to the second category, where the patients were subjected to significant puerperal infection risk factors. However, in the research where antibacterial sutures were used to close the fascia in the event of faecal peritonitis surgery (study population—104 patients), the antibacterial sutures proved highly effective. They reduced surgical infection by three times [12]. In our study in the investigation group, subcutaneous suturing was performed more frequently. Still, it is not considered a significant factor affecting the development of septic complications [3] and could not affect the outcomes of our study. The population of this study consisted of 165 female patients, with only 15 cases of endometritis and seven wound infections, which affected the quality of the statistical analysis. To verify this study’s findings, extensive, randomised, multi-centre research is required in the caesarean section population. 

During our study, the blood loss estimated during the operation that exceeded one litre did not increase the risk of developing septic complications, but the reduced level of haemoglobin on the third–fourth post-surgical day can be associated with the more frequent development of septic complications and, consequently, higher CRP. This explains why lower haemoglobin levels are associated with higher CRP levels. The lower the level of haemoglobin on the third–fourth day after surgery, the higher the risk of septic complications. The haemoglobin level of a patient before the operation is also essential. Even average blood loss in patients with presurgical anaemia reduces the blood haemoglobin to the level which causes hypo-perfusion of tissue and inhibits patient activation in the post-surgical period.

Consequently, routine healing of the wound is delayed. In this study, the haemoglobin level was not recorded before the operation. In an American study, the presurgical level of haemoglobin and haematocrit did not affect the frequency of endometritis development. Still, like in our research, a post-surgical lower level of haemoglobin and haematocrit was associated with the more frequent development of endometritis. The blood loss estimated during the operation could not be associated with more frequent endometritis [15]. In another study, a haemoglobin level below 11 g/dL was an independent risk factor for wound infection after caesarean section surgery [16]. Recording blood loss in a measuring tube during operation is not a sufficiently precise method [16,17] because it collects an unknown volume of amniotic fluid, and the risk of insufficient objectivity is high. 

It is widely believed that retained products of conception increase the risk of postpartum endometritis and bleeding. Therefore, cleaning the uterus with surgical swabs or gauze or rarely instrumentally with a curette after placental delivery is routine. Extensive discussions are caused by the fact that this procedure is not performed after vaginal birth, so there are no benefits to performing it during a caesarean section. Moreover, it only increases the risks of infection [18,19]. In our study, patients who underwent instrumental uterine revision during the caesarean section developed endometritis more frequently. This could be associated with basal endometrium traumatisation during this procedure, which promotes the implantation of microorganisms in the endometrium. It is also known that traumatising the basal endometrium of the pregnant uterus is a risk factor for developing Asherman’s syndrome [20]. The causes of impaired endometrial regeneration could include a low estrogen level in the mother’s blood, which decreases immediately after the end of pregnancy [20]. Berit et al. demonstrated that there was no clinical benefit of cervical dilatation and curettage in caesarean section but an increased time of surgery [21]. They did not find a significant influence of curettage on postpartum bleeding, necessity for blood transfusion, postoperative fever, endometritis, or wound infection. Berit et al. found statistically significantly prolonged operating time in patients undergoing cervical curettage compared to no curettage of the cervix in elective cesarean section [21]. During the postpartum and postabortion periods, curettage of the uterine cavity must be avoided as much as possible [20].

Stitching of the visceral and parietal peritoneum, muscles, or subcutaneous tissue was not a risk factor for septic postpartum complications. These findings are similar to the results of other studies [3,22,23]. The latest publications recommend suturing the uterus in one layer since, in this case, the risk of uterine rupture or wound dehiscence during the following pregnancies does not increase [24]. Suturing the visceral and parietal peritoneum is optional since it provides no short-term or long-term benefits. It only increases the duration of the surgery. In our study, suturing of the parietal peritoneum was associated with more muscular post-surgical pain. In the study of Eken et al., suturing of the visceral as well as parietal peritoneum was associated with more severe post-surgical pain and sympathetic response, which manifested as a higher frequency of heart rate, higher arterial blood pressure, and oliguria [25]. In another study, after the suturing of both peritoneal laminae, patients had a higher demand for analgesic medications [26]. The effect of peritoneal suturing on the development of adhesions is unclear because the results of studies are controversial [22,23,24]. Reports have shown that suturing of the rectus abdominis muscle creates higher post-surgical pain and the need for analgesic therapy [27]. Therefore, it is not recommended. This study failed to demonstrate that the suturing of the rectus abdominis muscle causes more severe pain in the post-surgical period. Probably, this is because the group of patients who had their muscles sutured included only six patients. Systematic reports, like this study, confirm that subcutaneous suturing does not affect the risk of developing septic complications [22,28].

The pain in excess-weight patients was stronger on the fourth post-surgical day, and their CRP levels were higher. In other studies, obese patients developed septic postpartum complications more frequently [29,30], which could explain the presence of more severe pain and higher levels of CRP. However, in this study, obese patients more frequently developed septic complications, but the difference is not statistically significant, probably due to the small study population.

The frequency of endometritis after emergency caesarean section surgery was 13.7%, and the frequency of wound infection was 5.3%. In the USA, these parameters are 3.8–11.7% and 2.4–4.5% [31,32]. The frequency of wound infections after scheduled surgery in our study was 3.9%, while in the USA—approximately 2% [33]. The frequency of wound infection after caesarean section, according to the definitions of USA Centers for Disease Control and Prevention (CDC) definitions, in Israel is 3.7% [34], in Egypt—5.3% [29], in Ethiopia—9.7% [35], at the Indian Tertiary Centre—10.3% [36]. The comparison of the frequency of septic complications in these studies is a complex process because the definitions of cases and the distribution of urgency degrees of caesarean sections in study populations are defined differently.

Nevertheless, it can be observed that the infection parameters in this research population are slightly higher than in other developed countries. In the population of our study, septic complications developed comparatively more frequently than in other developed countries. This could be associated with the effect of intraoperative factors, for instance, the performance of uterine curettage during surgery. Almost one-half of the patients in this study were subject to second-category caesarean section surgery, and more than two-thirds were emergency caesarean sections. By the recommendations of the American College of Obstetricians and Gynecologists (ACOG) [37,38], almost all these patients had indications for perioperative antibacterial prevention with added azithromycin. This could reduce the frequency of septic complications, but local recommendations did not provide for such tactics. In the study of Tita et al., in the USA, after the addition of azithromycin to antibacterial prevention therapy in the event of emergency caesarean section, the frequency of endometritis was reduced from 6.1% to 3.8%, while wound infection frequency—from 6.6% to 2.4% [32].

Research that would routinely determine the inflammation parameters in the mother’s blood after caesarean section cannot be found among studies published in international databases in the last ten years. This study performed postpartum US in 44% of the patients. It is believed that ultrasound images in postpartum endometritis are non-specific and may fail to differ from the US images of the uncomplicated postpartum period [39,40]. Routine detection of inflammatory parameters in blood and frequent performance of the US could likely cause hyperdiagnostics [41].

Wide variations in the procedures and techniques of caesarean section were observed in this study, where some factors differed from current recommendations. Several studies have been conducted where infection frequency was reduced in half after introducing evidence-based surgical techniques [42,43]. The septic complication parameters of this study’s population would decline after introducing unified evidence-based surgical tactics.

## 5. Conclusions

Antibacterial sutures during caesarean section do not affect the frequency of postpartum endometritis and surgical wound infection. To confirm this study’s findings, extensive randomised research is required in the caesarean section population. Instrumental revision of the uterine cavity during caesarean section surgery increases the risk of endometritis. The level of haemoglobin on the third–fourth post-surgical day and not the blood loss affects the development of septic postpartum complications. Suturing the parietal peritoneum increases pain in the post-surgical period but does not affect the outcome of infection.

## Figures and Tables

**Table 1:** Characteristics of this study’s population.

Parameter	Investigation Group (*n* = 67), *n* (%)	Control Group (*n* = 98), *n* (%)	*p*-Value
Maternal age, years ± SD	31.4 ± 5.4	32.7 ± 5.2	0.133
BMI, kg/m^2^	25.5 ± 5.7	24.4 ± 5.2	0.205
BMI ≥ 30 kg/m^2^	13 (19.4)	16 (16.3)	0.61
Smoking	2 (3)	4 (4.1)	1
Gestational diabetes	11 (16.4)	14 (14.3)	0.708
Nulliparous	36 (53.7)	50 (51.0)	0.732
Multiparous	31 (46.3)	48 (49.0)	
Gestation age, weeks	39.5 ± 2.0	39.7 ± 2.0	0.613
Preterm delivery	4 (6)	5 (5.1)	1
Positive group B streptococcus	13 (19.4)	10 (10.2)	0.235
In-labour caesarean section	32 (47.8)	45 (45.9)	0.816
Caesarean section in the second stage of labour	4 (6)	9 (9.2)	0.452
Duration of labour ≥ 12 h	9 (13.4)	9 (9.2)	0.403
Rupture of membranes before caesarean section	35 (52.2)	59 (60.2)	0.31
Number of vaginal examinations	2.9 ± 3.1	3.2 ± 3.1	0.59
Vaginal exams ≥ 5	24 (35.8)	31 (31.6)	0.575

SD—standard deviation; BMI—body mass index; *n*—number.

**Table 2:** Characteristics of the caesarean section.

Factor	Investigation Group (*n* = 67), *n* (%)	Control Group (*n* = 98), *n* (%)	*p*-Value
Operation time, minutes ± SD	37.2 ± 10.7	36.2 ± 9.9	0.539
Duration of caesarean section ≥ 1 h	3 (4.5)	2 (2)	0.397
Blood loss, mL	579.9 ± 134.6	585.7 ± 149.4	0.797
Blood loss ≥ 1000 mL	4 (6)	5 (5.1)	1
Closure of subcutaneous layer	53 (79.1)	60 (61.2)	0.015
Instrumental uterine cavity revision	11 (16.4)	12 (12.2)	0.447
Use of hemostatic sponge	7 (10.4)	13 (13.3)	0.586
Additional manipulations during caesarean section	9 (13.4)	8 (8.2)	0.274
Emergency caesarean section	39 (58.2)	71 (72.4)	0.057

SD—standard deviation; *n*—number.

**Table 3:** Parameters of the post-caesarean period.

Variable	Investigation Group (*n* = 67), *n* (%)	Control Group (*n* = 98), *n* (%)	*p*-Value
CRP, mg/L ± SD	64.4 ± 40.9	68.2 ± 39.1	0.544
Leukocytes, 10^3^/mm^3^	10.0 ± 2.8	9.3 ± 2.2	0.07
Postoperative haemoglobin, g/dL	11.3 ± 1.2	11.1 ± 1.3	0.323
Postoperative haemoglobin < 10.5 g/dL	15 (22.4)	34 (34.7)	0.089
Postpartum ultrasound	30 (44.8)	42 (42.9)	0.807
VAS score on 1st postoperative day	5.9 ± 1.9	5.9 ± 2.2	0.968
VAS score ≥ 5 on 1st postoperative day	47 (70.1)	63 (64.3)	0.433
VAS score on the 4th postoperative day	3.6 ± 1.4	3.4 ± 1.6	0.325
VAS score ≥ 5 on the 4th postoperative day	18 (26.9)	18 (18.4)	0.194
Duration of hospitalization after caesarean section, days	4.9 ± 2.1	4.7 ± 1.5	0.47
**At home**	*n* = 63	*n* = 83	-
VAS score	2.6 ± 2	2.7 ± 1.6	0.951
VAS score ≥ 5	11 (17.5)	11 (13.3)	0.482
Use of painkillers	19 (30.2)	24 (28.9)	0.87

CRP—C reactive protein; SD—standard deviation; VAS—Visual Analogue Scale; *n*—number.

**Table 4:** Septic outcomes in the post-caesarean period.

Outcomes	Investigation Group (*n* = 67), *n* (%)	Control Group (*n* = 98), *n* (%)	*p*-Value
**In hospital**	*n* = 67	*n* = 98	-
Endometritis	7 (10.4)	7 (7.1)	0.454
Wound infection	1 (1.5)	1 (1)	1
All septic complications	8 (11.9)	8 (8.2)	0.421
**At home**	*n* = 63	*n* = 83	-
Endometritis	3 (4.9)	0 (0)	0.08
Wound infection	3 (4.9)	2 (2.5)	0.451
Contacting a healthcare institution	8 (13.3)	6 (7.6)	0.266
Readmission	1 (1.6)	2 (2.5)	1
**Total**	*n* = 63	*n* = 83	-
Endometritis	8 (11.7)	7 (8.4)	0.401
Wound infection	4 (6.3)	3 (3.6)	0.444
Number of women with septic complications	10 (15.9)	10 (12)	0.506

*n*—number.

**Table 5:** The effect of intraoperative factors on the development of endometritis after caesarean section.

Factor	Endometritis in the Factor Group, *n* (%)	Endometritis in the Control Group, *n* (%)	*p*-Value
Duration of caesarean section ≥ 1 h, *n* = 5	2 (40)	13 (9.2)	0.082
Blood loss ≥ 1000 mL, *n* = 9	1 (11.1)	14 (10.2)	1
Instrumental uterine cavity revision, *n* = 21	5 (23.8)	10 (8)	0.043
Use of hemostatic sponge, *n* = 16	3 (18.8)	12 (9.2)	0.215
Additional manipulations during caesarean section, *n* = 12	1 (8.3)	12 (10.5)	1
Suturing of the visceral peritoneum, *n* = 11	0 (0)	15 (11.1)	0.605
Suturing of the parietal peritoneum, *n* = 20	1 (5)	14 (11.1)	0.694
Suturing of the rectus abdominis muscle, *n* = 6	0 (0)	15 (10.7)	1
Closure of subcutaneous layer, *n* = 100	12 (12)	3 (6.5)	0.39

*n*—number.

## Data Availability

All data were incorporated into the article.
